# Supplementary material for: Spatial super-spreaders and super-susceptibles in human movement networks
Source: Sci Rep. 2020 Oct 29;10:18642. doi: 10.1038/s41598-020-75697-z (PMC7596054; doi:10.1038/s41598-020-75697-z)
Supplement: Supplementary file 1 — Supplementary Information [file 41598_2020_75697_MOESM1_ESM.pdf]

Spatial super-spreaders and super-susceptibles in human movement networks

Wei Chien Benny Chin[1,+] and Roland Bouffanais[1,+,*]

[1]Singapore University of Technology and Design, 8 Somapah Road, Singapore 487372, Singapore
[*]bouffanais@sutd.edu.sg
[+]these authors contributed equally to this work

Supplementary Material

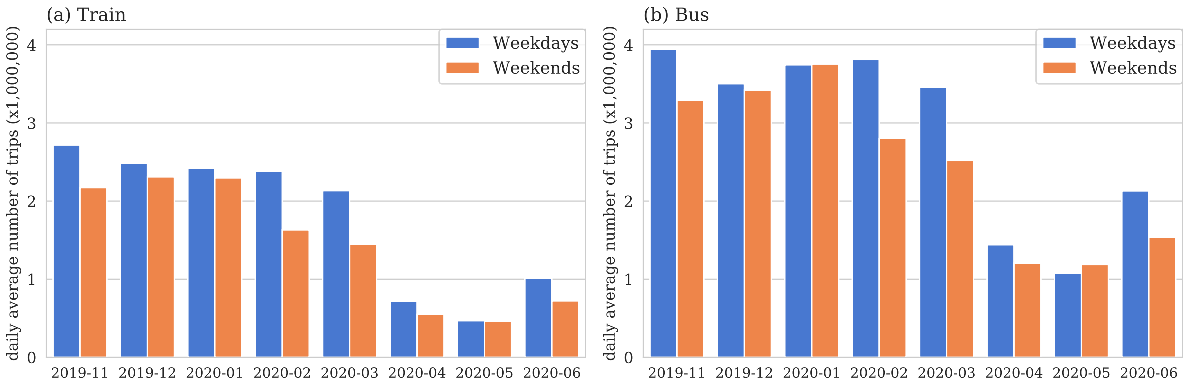

Figure S1. Daily average number of trips in each month from November 2019 to June 2020 in Singapore. Left: trips by train, right: trips by bus. Both sub-figures reveal a significant drop of trip counts during weekends in February and March 2019, while the weekday trips remain similar as before; both weekdays and weekends have a low number of trips in April and May 2020, when the whole country of Singapore was under the so-called “Circuit Breaker" measure (partial country lockdown with only essential movements allowed); the Circuit Breaker phase ended on 1st of June 2020, but most people were advised to continue “working from home." In this study, we focused on the first three months (November 2019 to January 2020) because the human flow activity was relatively stable and consistent, thereby reflecting the regular movement patterns; starting from February 2020, the movement patterns were changed following the onset of the Covid-19 pandemics, which has been affected by various unknown factors.

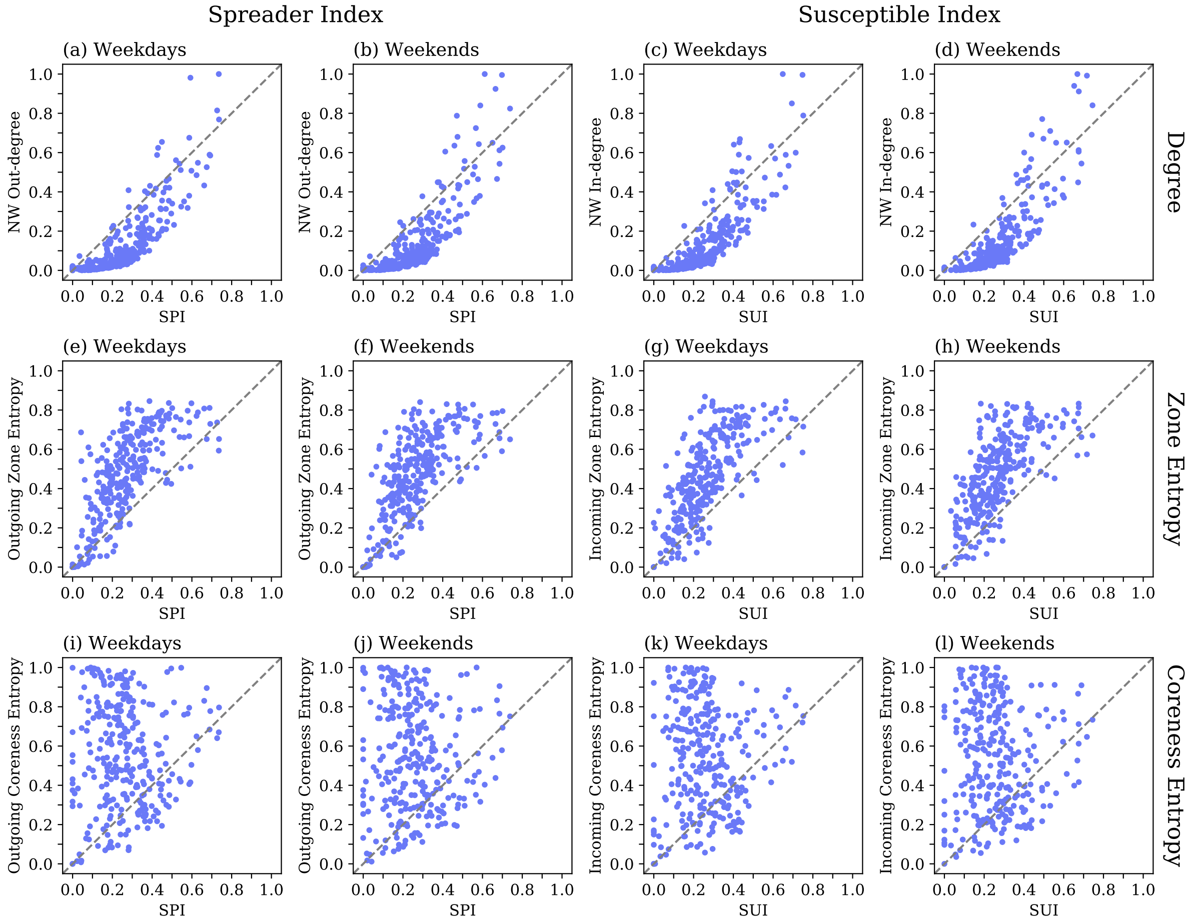

Figure S2. **Comparison between the two indexes (*SPI* and *SUI*) and the three components (weighted degree, zone entropy, and coreness entropy) for weekdays and weekends.** First column: *SPI* for weekdays, second column: *SPI* for weekends, third column: *SUI* for weekdays, fourth column: *SUI* for weekends. First row: normalized weighted degree, second row: zone entropy, third row: coreness entropy.

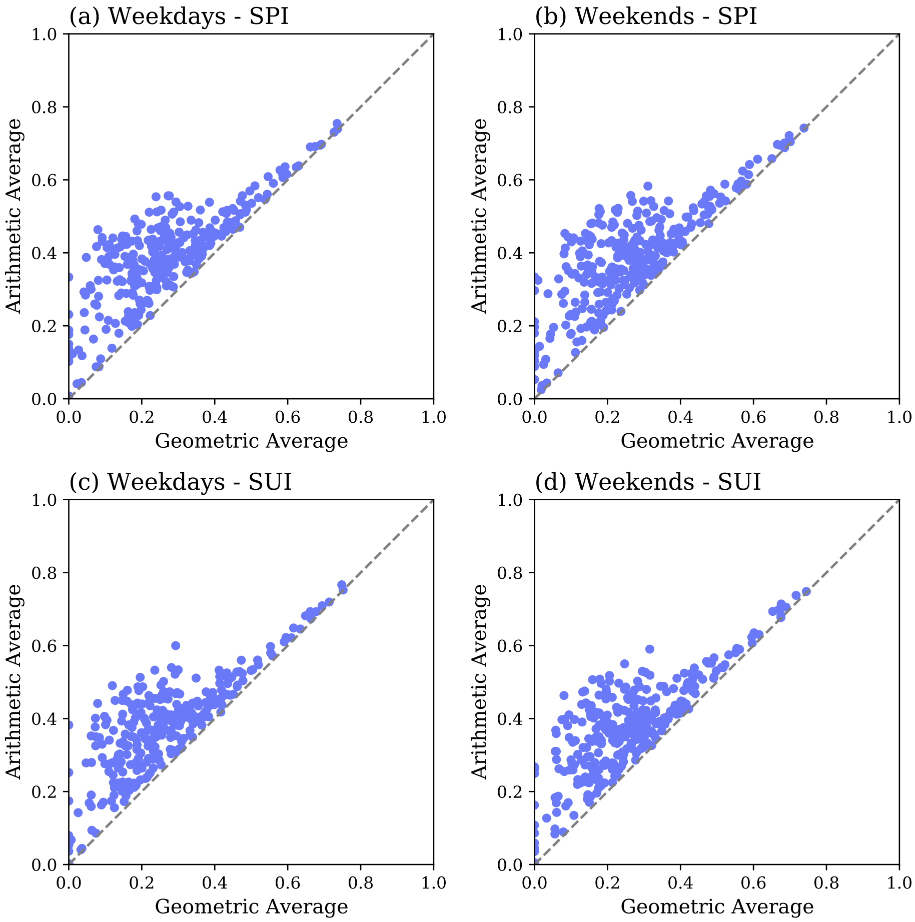

Figure S3. Comparison between geometric average and arithmetic average versions of *SPI* and *SUI*. Left column: weekdays, right column: weekends. Top row: *SPI*, bottom row: *SUI*.

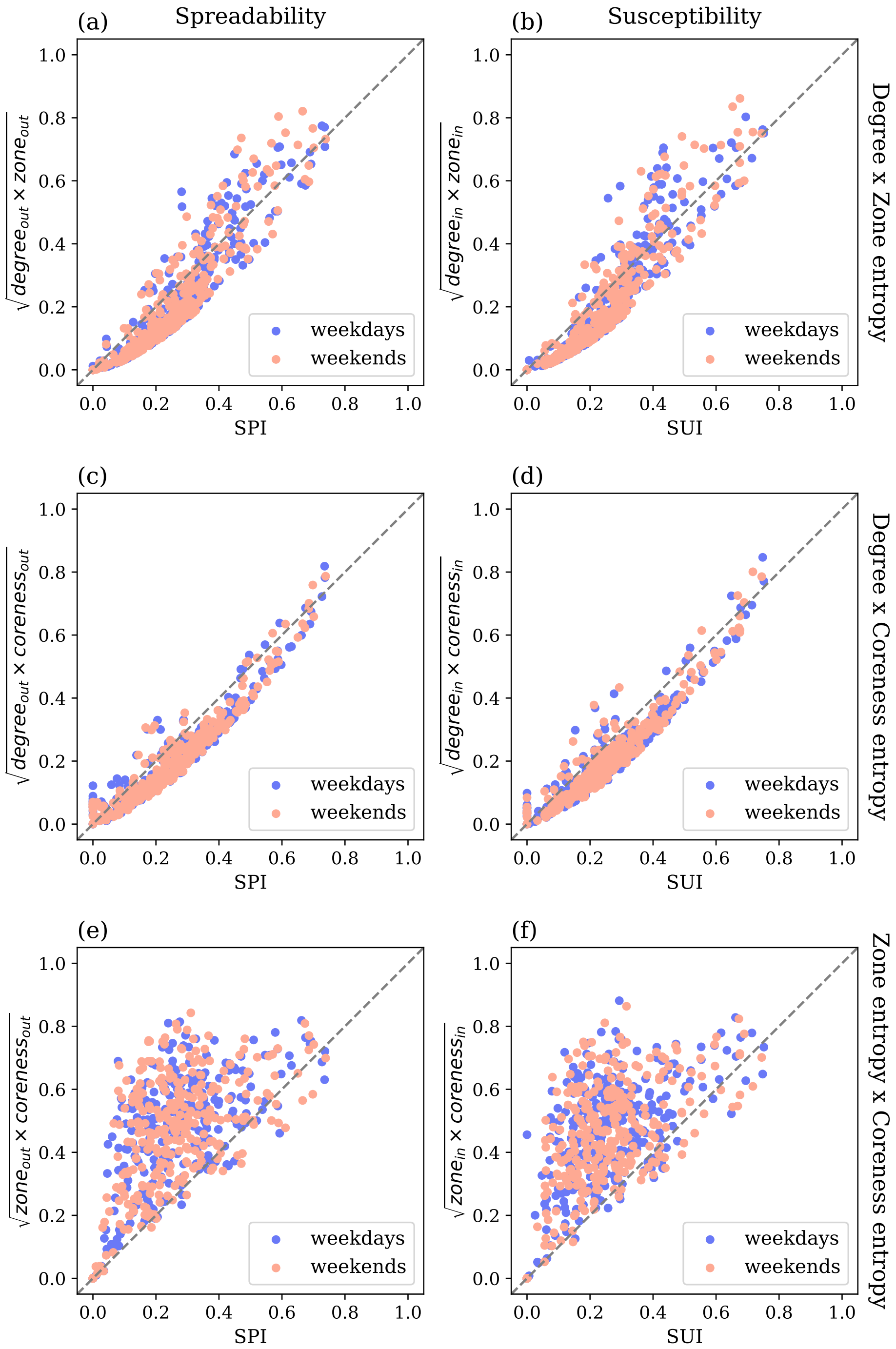

Figure S4. Comparison between geometric average of two components and the corresponding SPI or SUI index. Left column: SPI, right column: SUI. Top row: geometric average of degree and zone-entropy, middle row: geometric average of degree and coreness-entropy, bottom row: geometric average of zone-entropy and coreness-entropy. Blue dots: weekdays, pink dots: weekends.

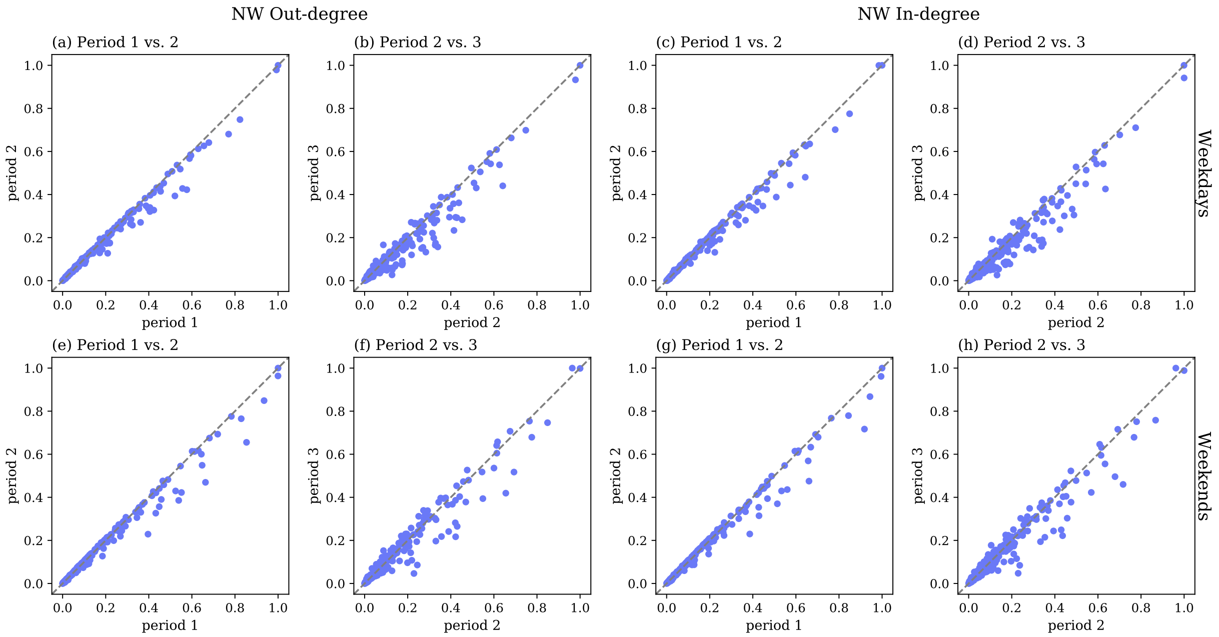

Figure S5. Comparison of normalized weighted degrees between the three periods. Period 1: December 2019–January 2020; period 2: February–March 2020; period 3: April–May 2020. Two columns on the left: out-degree, two columns on the right: in-degree. Top row: weekdays, bottom row: weekends.

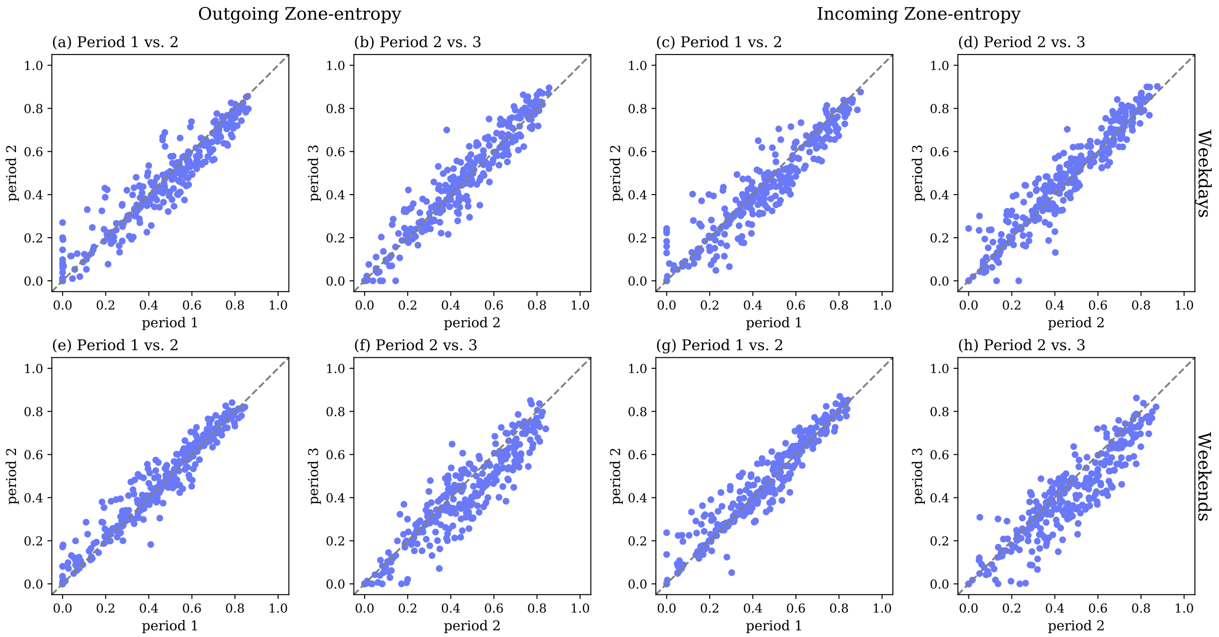

Figure S6. Comparison of zone-entropy between the three periods. Period 1: December 2019– January 2020; period 2: February–March 2020; period 3: April–May 2020. Two columns on the left: outgoing neighbors, two columns on the right: incoming neighbors. Top row: weekdays, bottom row: weekends.

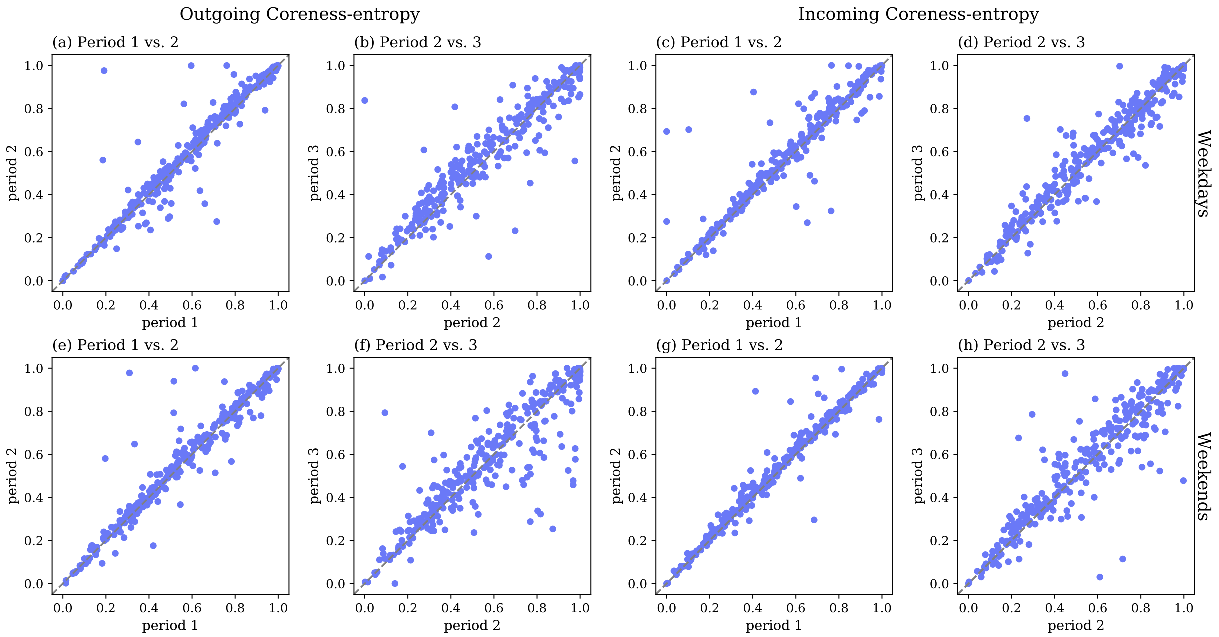

Figure S7. Comparison of coreness-entropy between the three periods. Period 1: December 2019–January 2020; period 2: February–March 2020; period 3: April–May 2020. Two columns on the left: outgoing neighbors, two columns on the right: incoming neighbors. Top row: weekdays, bottom row: weekends.

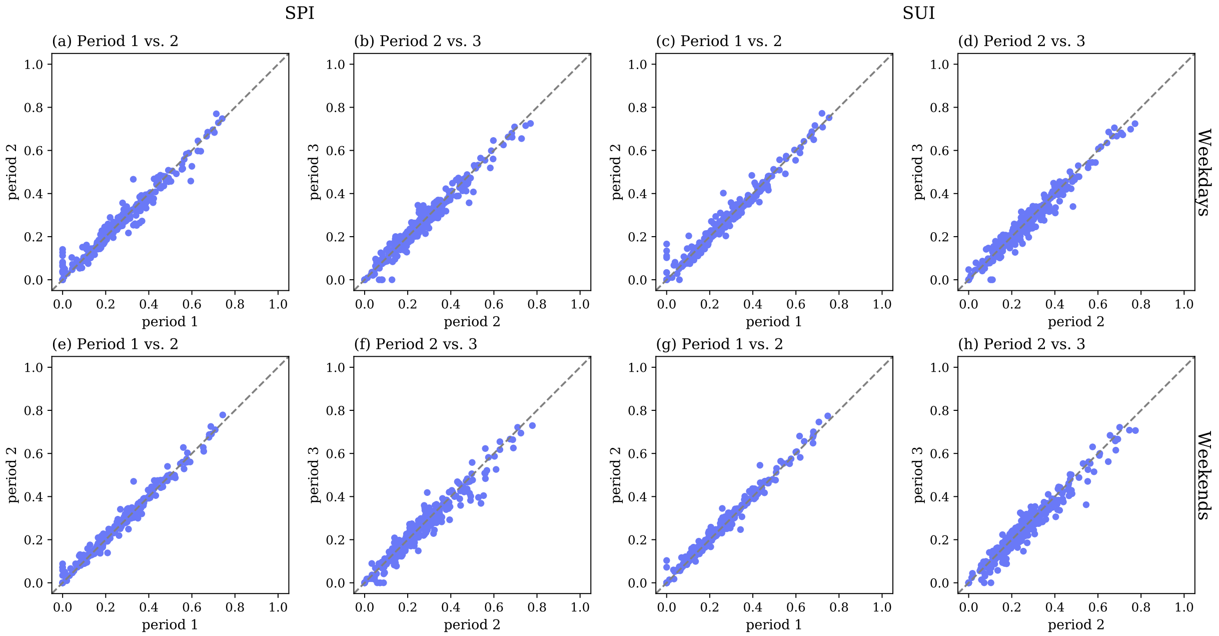

Figure S8. Comparison of *SPI* or *SUI* between the three periods. Period 1: December 2019–January 2020; period 2: February–March 2020; period 3: April–May 2020. Two columns on the left: *SPI*, two columns on the right: *SUI*. Top row: weekdays, bottom row: weekends.

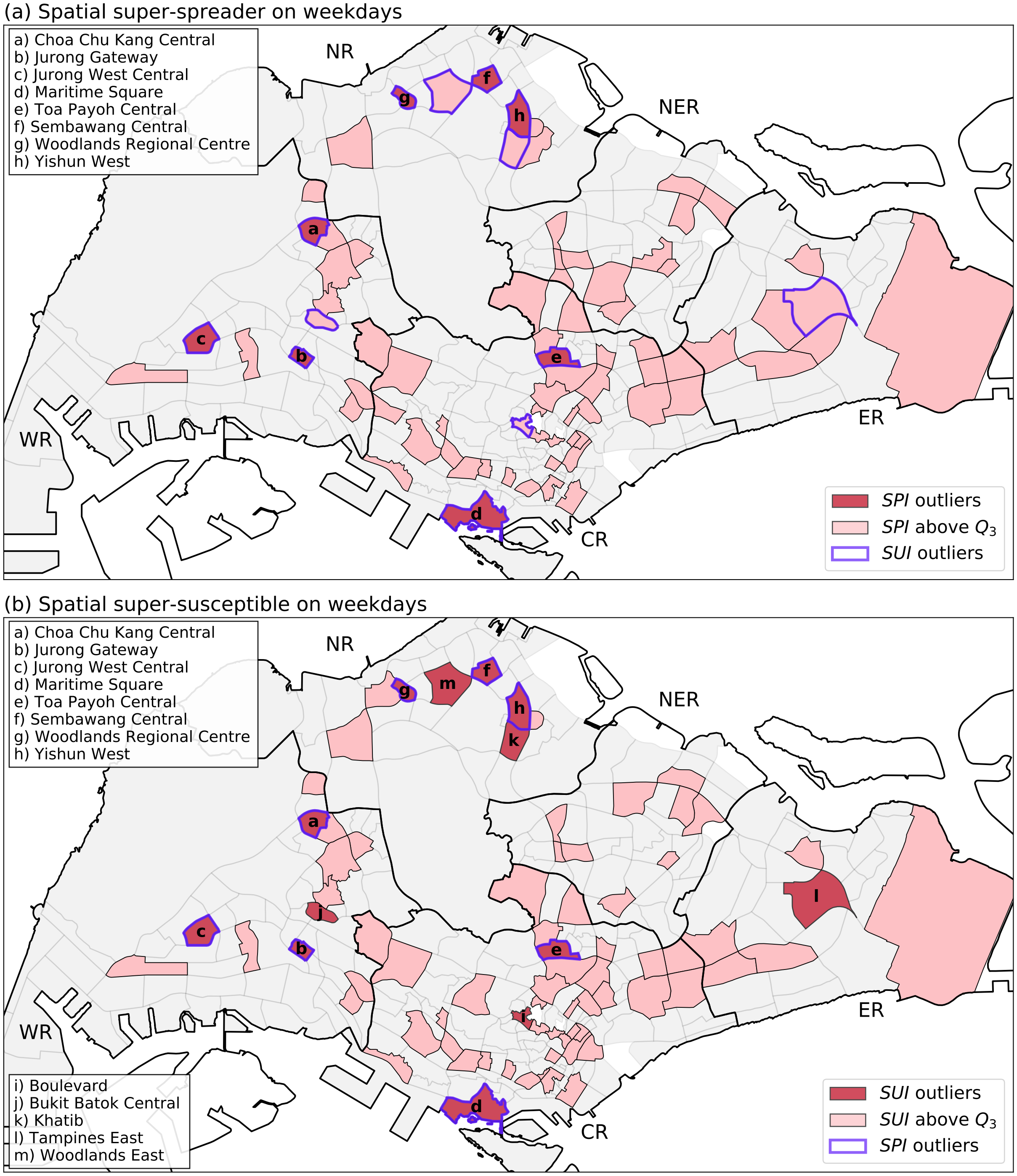

Figure S9. Spatial distribution of (a) spreader index (SPI), and (b) susceptible index (SUI) for weekdays of period 1 (December 2019–January 2020). The subzones with purple border in (a) and (b) respectively indicate the spatial super-susceptibles ($SUI \geq Q_3 + 1.5 \times IQR$) and spatial super-spreader ($SPI \geq Q_3 + 1.5 \times IQR$). (generated with Python (3.7.5), Matplotlib (3.2.1) and GeoPandas (0.7.0))

(a) Spatial super-spreader on weekdays

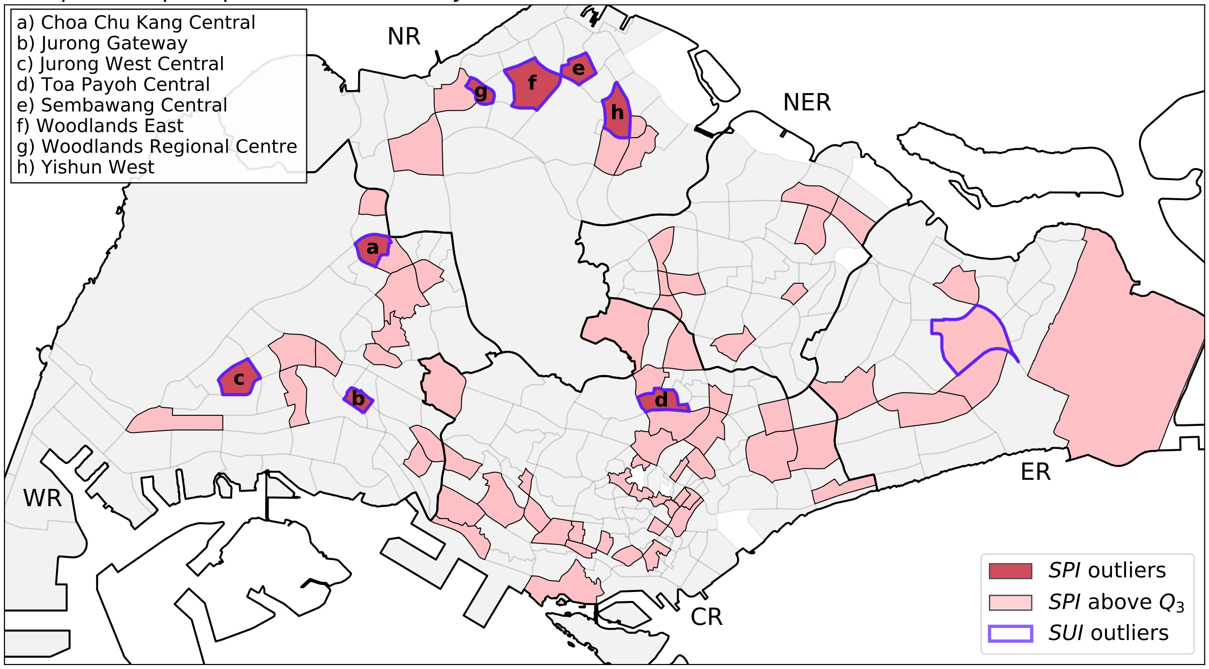

(b) Spatial super-susceptible on weekdays

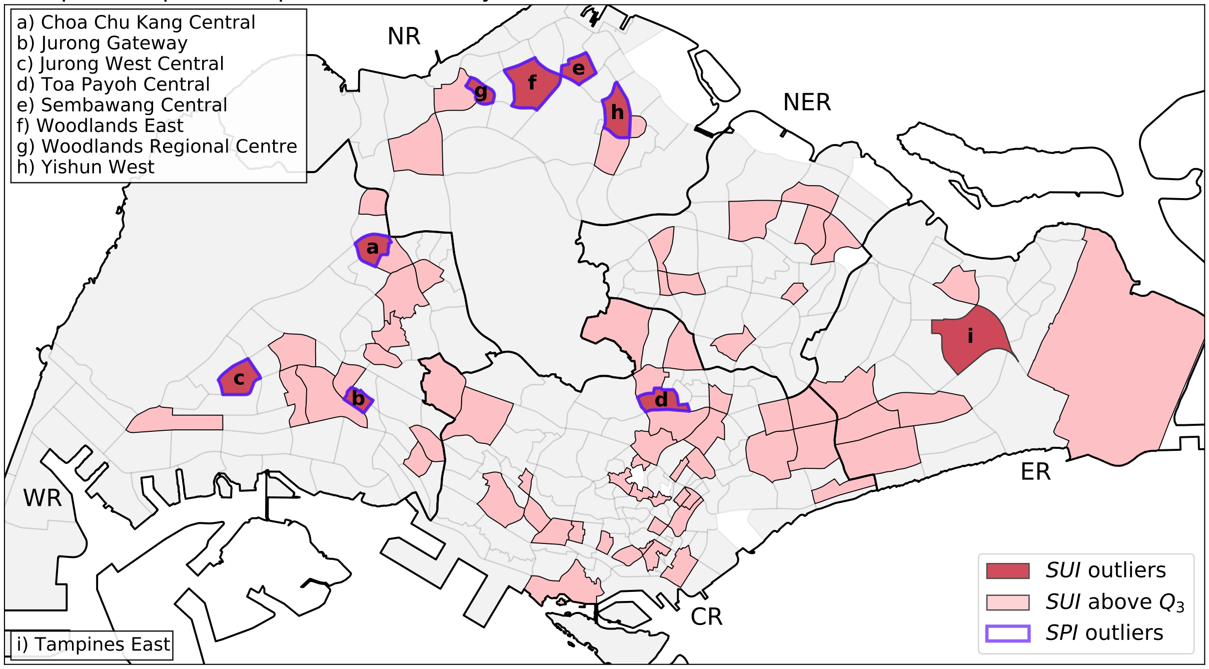

Figure S10. Spatial distribution of (a) spreader index (*SPI*), and (b) susceptible index (*SUI*) for weekdays of period 2 (February–March 2020). The subzones with purple border in (a) and (b) respectively indicate the spatial super-susceptibles ($SUI \geq Q_3 + 1.5 \times IQR$) and spatial super-spreader ($SPI \geq Q_3 + 1.5 \times IQR$). (generated with Python (3.7.5), Matplotlib (3.2.1) and GeoPandas (0.7.0))

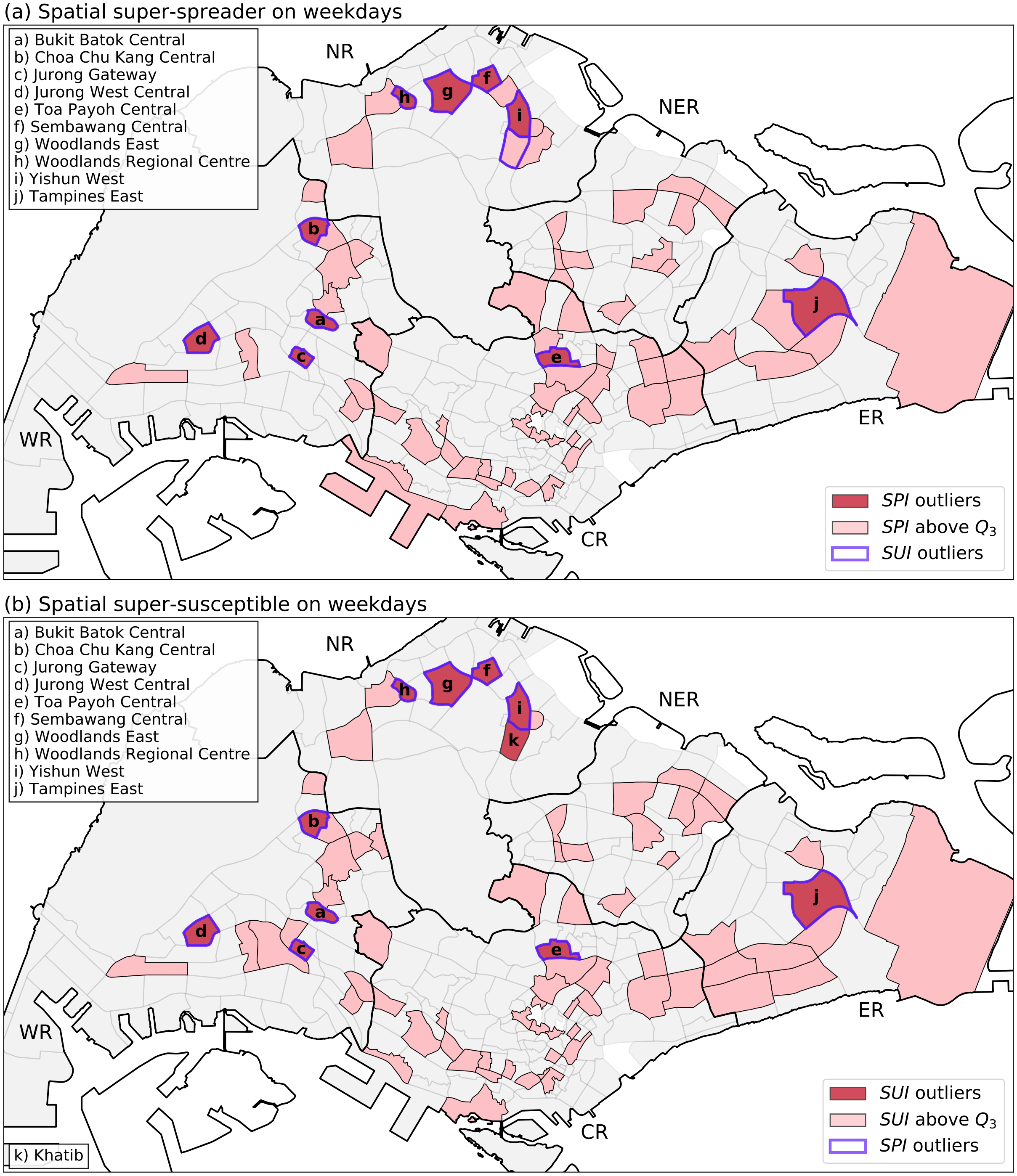

Figure S11. **Spatial distribution of (a) spreader index (SPI), and (b) susceptible index (SUI) for weekdays of period 3 (April–May 2020).** The subzones with purple border in (a) and (b) respectively indicate the spatial super-susceptibles ($SUI \geq Q_3 + 1.5 \times IQR$) and spatial super-spreader ($SPI \geq Q_3 + 1.5 \times IQR$). (generated with Python (3.7.5), Matplotlib (3.2.1) and GeoPandas (0.7.0))

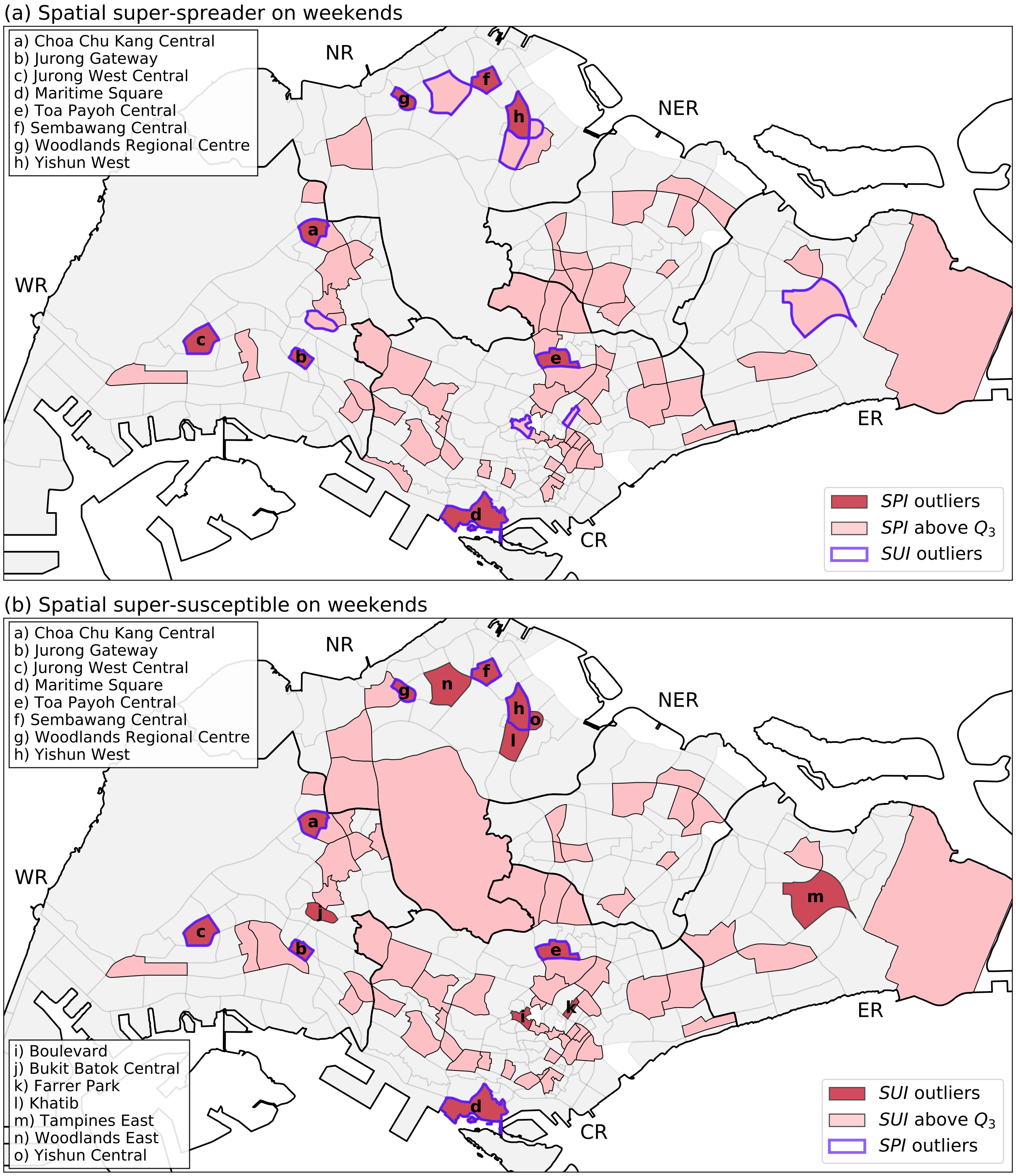

Figure S12. Spatial distribution of (a) spreader index (SPI), and (b) susceptible index (SUI) for weekends of period 1 (December 2019–January 2020). The subzones with purple border in (a) and (b) respectively indicate the spatial super-susceptibles ($SUI \geq Q_3 + 1.5 \times IQR$) and spatial super-spreader ($SPI \geq Q_3 + 1.5 \times IQR$). (generated with Python (3.7.5), Matplotlib (3.2.1) and GeoPandas (0.7.0))

(a) Spatial super-spreader on weekends

(b) Spatial super-susceptible on weekends

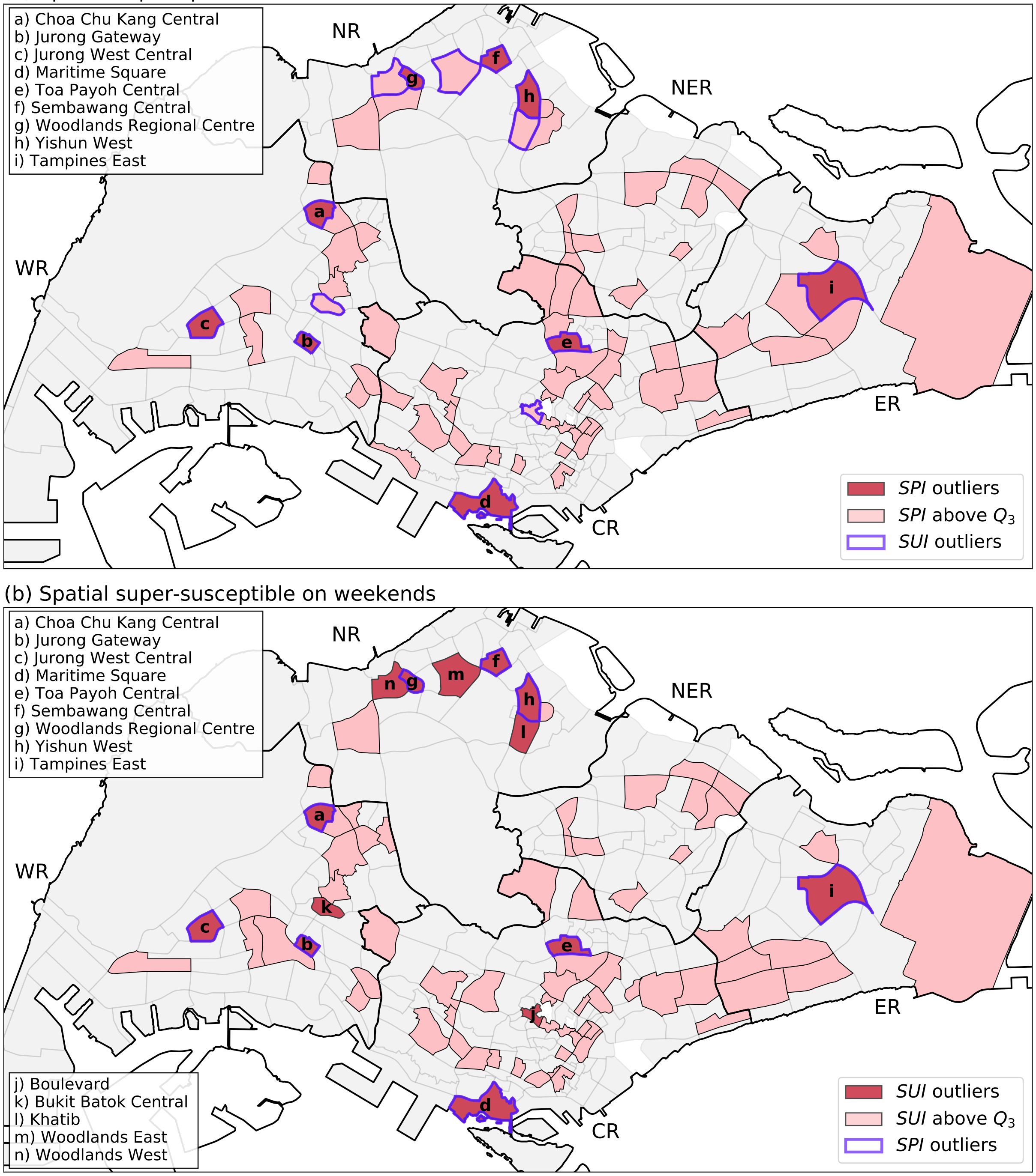

Figure S13. Spatial distribution of (a) spreader index (SPI), and (b) susceptible index (SUI) for weekends of period 2 (February–March 2020). The subzones with purple border in (a) and (b) respectively indicate the spatial super-susceptibles ($SUI \geq Q_3 + 1.5 \times IQR$) and spatial super-spreader ($SPI \geq Q_3 + 1.5 \times IQR$). (generated with Python (3.7.5), Matplotlib (3.2.1) and GeoPandas (0.7.0))

(a) Spatial super-spreader on weekends

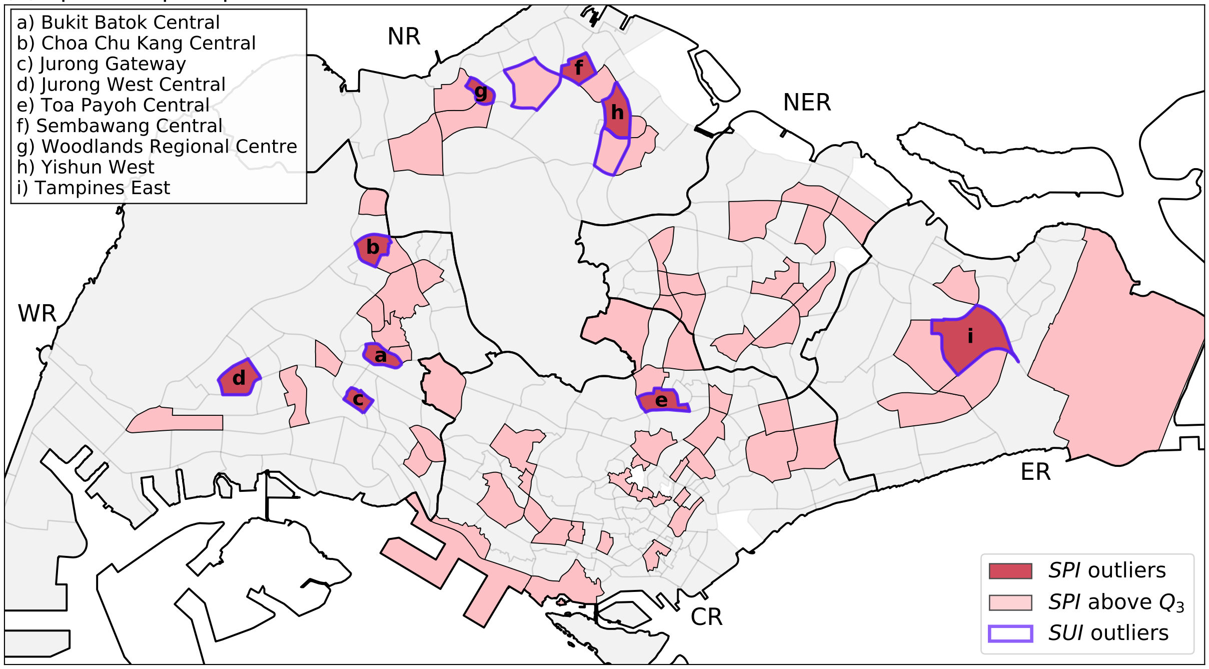

(b) Spatial super-susceptible on weekends

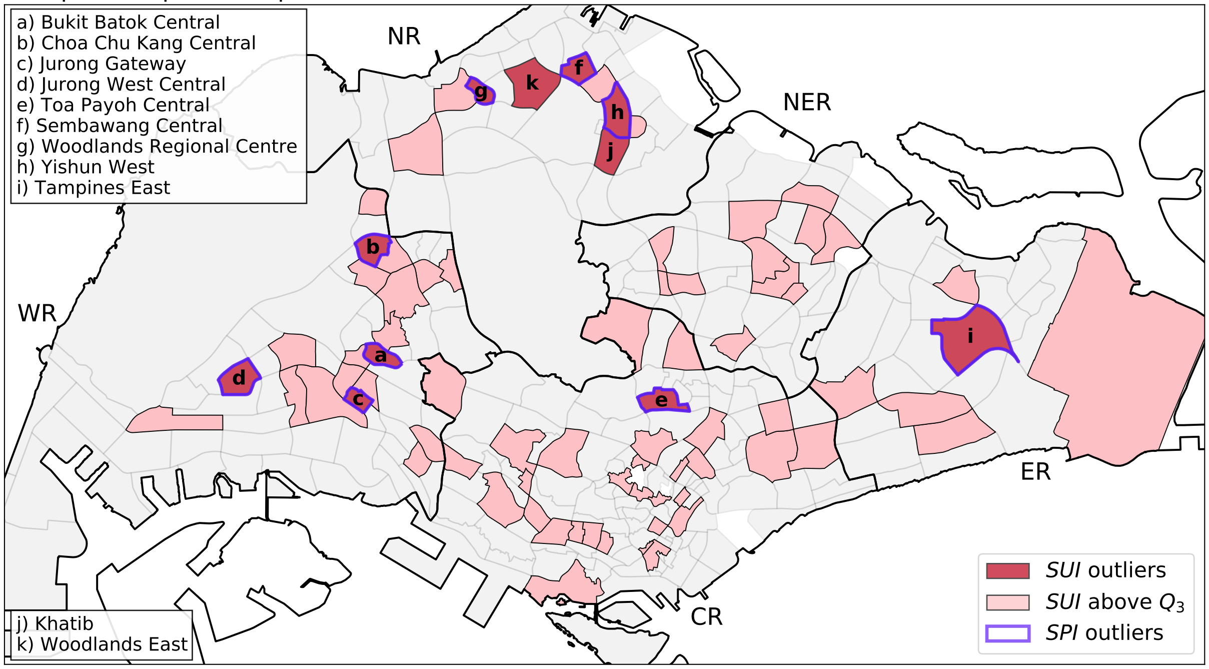

Figure S14. Spatial distribution of (a) spreader index (SPI), and (b) susceptible index (SUI) for weekends of period 3 (April–May 2020). The subzones with purple border in (a) and (b) respectively indicate the spatial super-susceptibles ($SUI \geq Q_3 + 1.5 \times IQR$) and spatial super-spreader ($SPI \geq Q_3 + 1.5 \times IQR$). (generated with Python (3.7.5), Matplotlib (3.2.1) and GeoPandas (0.7.0))
